# Acetyl-cinobufagin suppresses triple-negative breast cancer progression by inhibiting the STAT3 pathway

**DOI:** 10.18632/aging.204967

**Published:** 2023-08-28

**Authors:** Yufeng Qi, Haodong Wu, Tianru Zhu, Zitian Liu, Conghui Liu, Congzhi Yan, Zhixuan Wu, Yiying Xu, Ying Bai, Lehe Yang, Dezhi Cheng, Xiaohua Zhang, Haiyang Zhao, Chengguang Zhao, Xuanxuan Dai

**Affiliations:** 1 The First People’s Hospital of Xiaoshan District, Xiaoshan Affiliated Hospital of Wenzhou Medical University, Hangzhou 311200, Zhejiang, China; 2 The First Affiliated Hospital, Wenzhou Medical University, Wenzhou 325000, Zhejiang, China; 3 Institute of Life Sciences, Biomedical Collaborative Innovation Center of Zhejiang Province, Wenzhou University, Wenzhou 325035, Zhejiang, China; 4 School of Pharmaceutical Sciences, Wenzhou Medical University, Wenzhou 325035, Zhejiang, China

**Keywords:** acetyl-cinobufagin, STAT3, epithelial-mesenchymal transition, STAT3 inhibitor, triple negative breast cancer

## Abstract

Background: The incidence of breast cancer (BC) worldwide has increased substantially in recent years. Epithelial-mesenchymal transition (EMT) refers to a crucial event impacting tumor heterogeneity. Although cinobufagin acts as an effective anticancer agent, the clinical use of cinobufagin is limited due to its strong toxicity. Acetyl-cinobufagin, a pre-drug of cinobufagin, was developed and prepared with greater efficacy and lower toxicity.

Methods: A heterograft mouse model using triple negative breast cancer (TNBC) cell lines, was used to evaluate the potency of acetyl-cinobufagin. Signal transducer and stimulator of transcription 3 (STAT3)/EMT involvement was investigated by gene knockout experiments using siRNA and Western blot analysis.

Results: Acetyl-cinobufagin inhibited proliferation, migration, and cell cycle S/G2 transition and promoted apoptosis in TNBC cells *in vitro*. In general, IL6 triggered the phosphorylation of the transcription factor STAT3 thereby activating the STAT3 pathway and inducing EMT. Mechanistically, acetyl-cinobufagin suppressed the phosphorylation of the transcription factor STAT3 and blocked the interleukin (IL6)-triggered translocation of STAT3 to the cell nucleus. In addition, acetyl-cinobufagin suppressed EMT in TNBC by inhibiting the STAT3 pathway. Experiments in an animal model of breast cancer clearly showed that acetyl-cinobufagin was able to reduce tumor growth.

Conclusions: The findings of this study support the potential clinical use of acetyl-cinobufagin as a STAT3 inhibitor in TNBC adjuvant therapy.

## INTRODUCTION

The most frequent malignancy in women globally is breast cancer (BC). In 2020, there were 2,261,419 new patients, or 11.7% of total cancer cases and 24.5% of female tumors, based on the figures from IARC/WHO. Additionally, 6.9% of all cancers and 15.5% of female tumors, or 684,996 deaths, were projected to be caused by BC [1]. Triple-negative breast cancer (TNBC) has a lower survival rate and is more likely to recur and metastasize when compared to other subtypes of BC. TNBC has fewer therapeutic choices than other forms of invasive breast cancer because of the absence of these specific targets (HR and HER2), and the current treatment regimens, which include surgery, chemotherapy, and radiotherapy, are insufficient [2]. Finding novel molecular medicines with great efficacy and minimal toxicity is therefore crucial.

A member of the STAT protein family called Signal Transducer and Stimulator of Transcription 3 (STAT3) controls several genes [3, 4]. STAT3 encourages the growth of cancer cells, immunological suppression, angiogenesis, the spread of metastatic disease, and the emergence of therapeutic resistance [5–7]. Many studies have revealed that STAT3 also plays crucial roles in BC development, as well as in drug resistance development in BC patients receiving chemotherapy [8–10]. The regulation of STAT3 activity could effectively suppress carcinogenesis, cancer development, and invasive behavior in TNBC [11]. As a result, STAT3 is now acknowledged as a viable molecular target for the creation of a successful treatment for TNBC.

The phenotypic transformation of epithelial cells into mesenchymal cells is known as the epithelial-mesenchymal transition (EMT) [12]. EMT has been linked to tumor development and metastasis in TNBC, according to several earlier investigations [13, 14]. Increasing evidence indicates that various treatment regimens inhibit migratory behavior by downregulating EMT [15]. In recent years, research has shown that interleukin 6 (IL6) induces EMT of cancer cells by STAT3 activation [16–18]. At the same time, STAT3 inhibitors can suppress cancer development, invasive activity, and migratory behavior through inhibition of EMT [19].

Cinobufagin is a key active constituent of Chansu [20]. Cinobufagin has been suggested to possess significant anticancer activity [21, 22]. However, although cinobufagin possesses satisfactory cytotoxic activity against a variety of cancer cells *in vitro*, its toxicity and severe side effects following its administration *in vivo* are undesirable, clearly revealing the limitations of the clinical use of cinobufagin. In addition, its rapid metabolism, insolubility, and short half-life, further restrict clinical application [23]. In this study, acetyl-cinobufagin, a cinobufagin pre-drug, was created and manufactured by aspirin and diacerein standards to produce new analogs with increased potency and decreased toxicity. Yang et al. have applied this processing method to bufalin and obtained good results [24]. Acetyl-cinobufagin contributes to improving drug administration and might enhance the anticancer effect.

## RESULTS

### Acetyl-cinobufagin inhibits the viability and proliferation of TNBC cells

**Figure 1:**
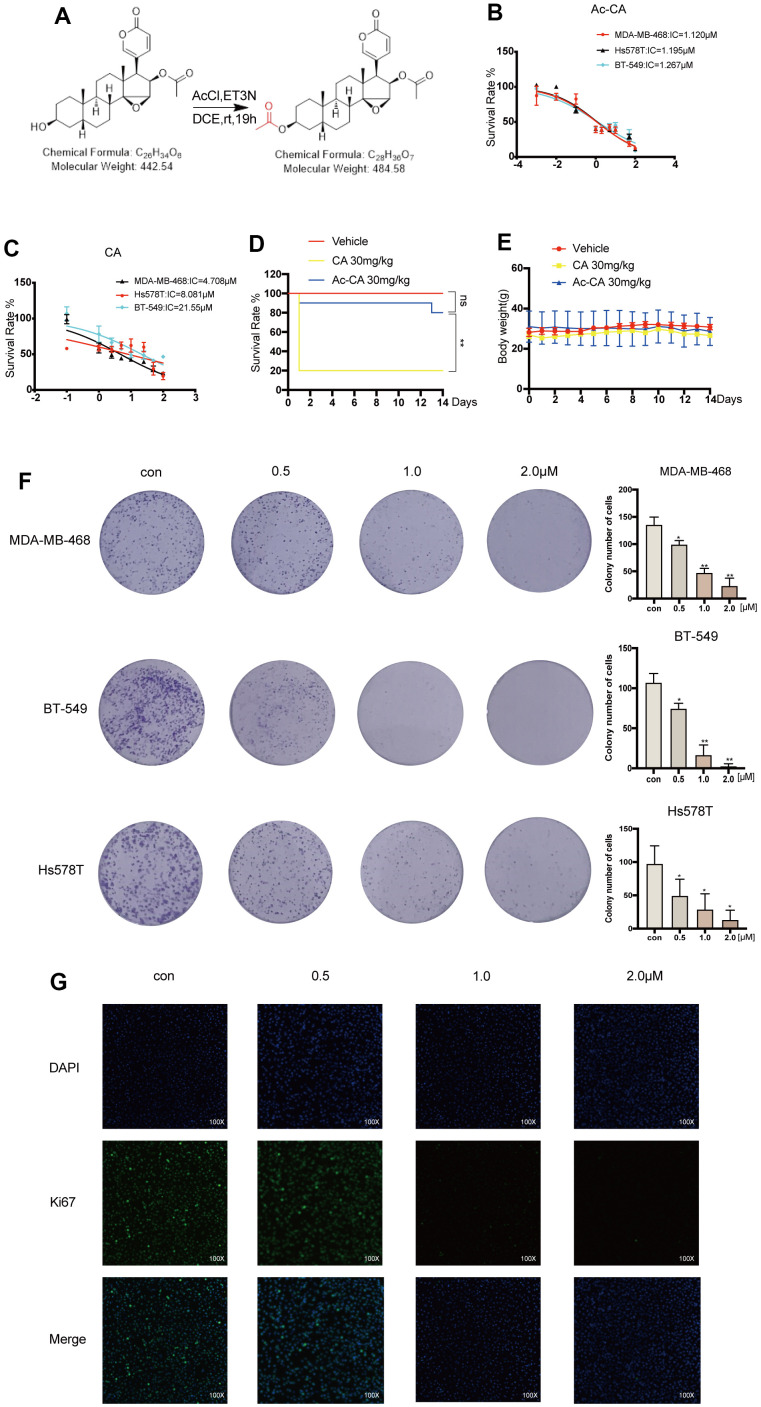
**Acetyl-cinobufagin inhibits the proliferation of TNBC cells.** (**A**) Chemical composition of acetyl-cinobufagin. (**B**, **C**) The suppression rate was determined by the MTT assay in MDA-MB-468, BT-549, Hs578T cells. (**D**) The cinobufagin and acetyl-cinobufagin groups were intraperitoneally injected with 30 mg/kg on the 1^st^ day, and the death rate of the animals were recorded for 14 d. (**E**) Body weight curve of the animals. (**F**) Colony forming analysis was completed with the aforementioned three cell lines with/without acetyl-cinobufagin treatment. (**G**) Changes in the signals received by proteins (Ki67) in BT-549 cells were analyzed by immunofluorescence staining. Statistical analysis is illustrated as follows: (**D**) Log-rank (Mantel-Cox) test; (**F**) t-test. *p < 0.05; **p < 0.01.

The pre-drug of cinobufagin, acetyl-cinobufagin, was created and manufactured to completely understand the metabolic impact of cinobufagin and seek novel analogs with higher efficacy and reduced toxicity (Figure 1A). The MTT test was used to measure the longevity of cells to assess the effectiveness of cinobufagin and acetyl-cinobufagin on human BC cell lines. The results revealed that the IC_50_ of acetyl-cinobufagin against MDA-MB-468, BT-549 and Hs578T cells were 1.120, 1.267 and 1.195 μM, respectively (Figure 1B); while IC_50_ of cinobufagin against MDA-MB-468, BT-549 and Hs578T were 4.708, 21.550 and 8.081 μM, respectively (Figure 1C). In addition, the IC_50_ of acetyl-cinobufagin against MDA-MB-231 was 13.86 μM, respectively (Supplementary Figure 1A). Meanwhile, the IC_50_ of acetyl-cinobufagin against a normal breast cell line (MCF-10A) was 35.90 μM (Supplementary Figure 1B). To determine whether the chemicals were safe, sudden toxicity tests were performed. Only a few (2/10) of the animals in the cinobufagin group died after receiving the injection of acetyl-cinobufagin, compared to the majority (8/10) of the animals in the cinobufagin group (Figure 1D). The body weight of the animals in the acetyl-cinobufagin group remained constant in contrast to the negative control (vehicle) group (Figure 1E). Additionally, acetyl-cinobufagin greatly decreased the capacity of the TNBC cell lines to form colonies (Figure 1F, and Supplementary Figure 1C). The evaluation of the effect of acetyl-cinobufagin on the proliferative activity of TNBC cells through immunofluorescence analysis of the expression of Ki67 in BT-549 cells revealed that the fluorescence intensity of Ki67 was markedly dose-dependently attenuated (Figure 1G), indicating that acetyl-cinobufagin suppresses cell growth and proliferation in a dosage-dependent manner.

### Acetyl-cinobufagin inhibits the migration of TNBC cells

**Figure 2:**
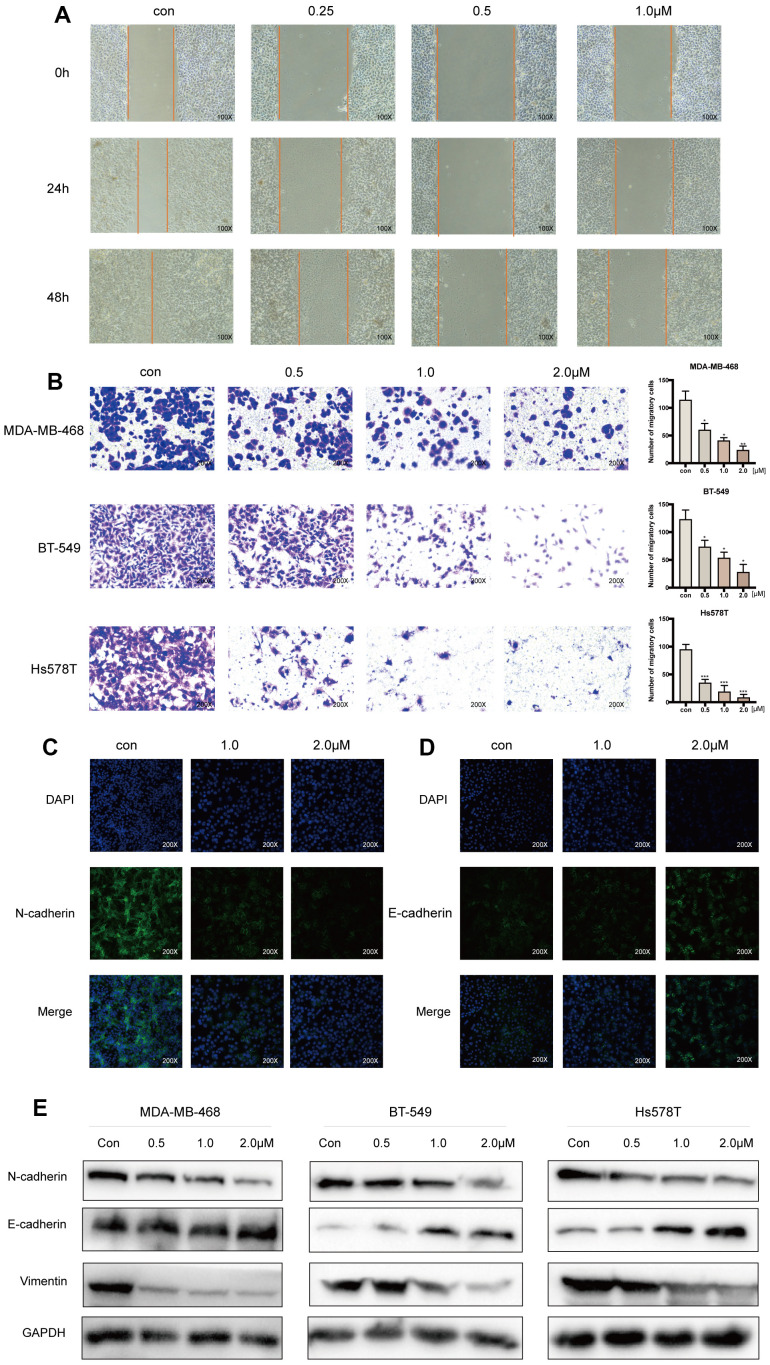
**Acetyl-cinobufagin inhibits the migration of TNBC cells.** (**A**) Wound healing assay was performed using the BT-549 cells with/without acetyl-cinobufagin treatment. (**B**) Transwell migration assay was performed with the MDA-MB-468, BT-549 and Hs578T cells. (**C**, **D**) Changes in the expression of EMT marker proteins (E-cadherin and N-cadherin) in Bt-549 cells were verified by immunofluorescence staining. (**E**) Expression of proteins related to the EMT signaling pathways as identified by Western blotting. *p < 0.05; **p < 0.01; ***p < 0.001 compared to the control as per Student’s t-test.

Acetyl-cinobufagin dose-dependently and significantly suppressed the migration of the TNBC cell lines MDA-MB-468, BT-549, and Hs578T, according to an examination of the hindering impact of the drug using the wound healing test (Figure 2A, and Supplementary Figure 1C, 1D). Additionally, the Transwell test we used to assess the inhibitory effect of acetyl-cinobufagin on the migration of the TNBC cell lines MDA-MB-468, BT-549, and Hs578T revealed that the drug significantly inhibited their migration in a dose-dependent manner (Figure 2B). We used immunofluorescence labeling to examine their expression in the TNBC cells because the hallmark of EMT is the increase of the protein N-cadherin expression followed by the decrease of the protein E-cadherin expression. According to the immunofluorescence analysis, acetyl-cinobufagin administration raised the expression of E-cadherin while decreasing the expression of the protein N-cadherin (Figure 2C, 2D), indicating an inhibitory effect on EMT. Western blot examination demonstrated increased expression of E-cadherin and decreased expression of vimentin in the presence of acetyl-cinobufagin compared to the control group, suggesting a similar effect of acetyl-cinobufagin on the expression of EMT marker proteins (Figure 2E). Together, our results showed that acetyl-cinobufagin not only prevented TNBC cells from migrating and proliferating but also controlled the production of a protein necessary for migration.

### Acetyl-cinobufagin promotes apoptosis of TNBC cells

**Figure 3:**
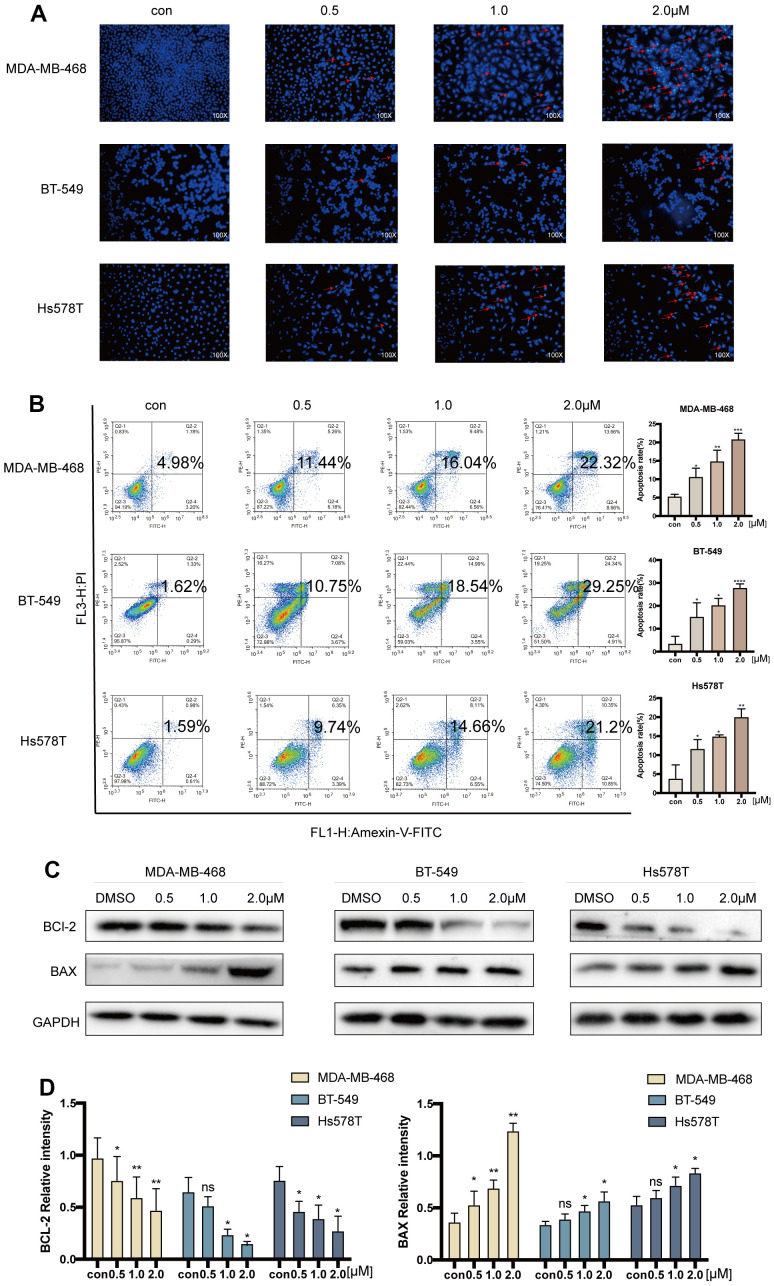
**Acetyl-cinobufagin induced cell apoptosis in TNBC cells.** (**A**) Hoechst 33342 staining and (**B**) flow cytometric analysis were performed to study the cell apoptosis rate after treatment with 0, 0.5, 1.0, and 2.0 μM of acetyl-cinobufagin for 24 h in MDA-MB-468, BT-549 and Hs578T cells by Annexin V and PI. (**C**, **D**) The expression levels of BAX and BCL-2 in above-mentioned three TNBC cell lines were determined by immunoblotting after treatment with acetyl-cinobufagin at three concentrations for 24 h. *p < 0.05; **p < 0.01; ***p < 0.001; ****p < 0.0001 compared with the control using the Student’s t-test.

The effect of various concentrations of acetyl-cinobufagin (0, 0.5, 1.0, and 2.0 μM) on apoptosis in the TNBC cell lines MDA-MB-468, BT-549 and Hs578T was analyzed by Hoechst 33342 staining of apoptotic cell nuclei. The results showed that cells treated with acetyl-cinobufagin exhibited higher nuclear staining with Hoechst 33342 (blue fluorescence) indicating the induction of substantial apoptosis (Figure 3A). A similar apoptotic effect of acetyl-cinobufagin on these 3 TNBC cell lines was observed by flow cytometry analysis, after treating these cells with acetyl-cinobufagin for 24 h. Apoptotic cells were favorably marked with Annexin V, either by itself or in combination with PI. The dead cells, however, were just PI-positive. Acryl-cinobufagin greatly raised the percentage of apoptotic cells, according to the data (Figure 3B). The outcomes additionally indicated that acetyl-cinobufagin dose-dependently regulated the expression of apoptosis-associated proteins, specifically increasing BAX expression while decreasing BCL2 expression (Figure 3C, 3D). In TNBC cells, acetyl-cinobufagin dramatically increased apoptosis.

### Acetyl-cinobufagin inhibits S/G2 transition in TNBC cells

**Figure 4:**
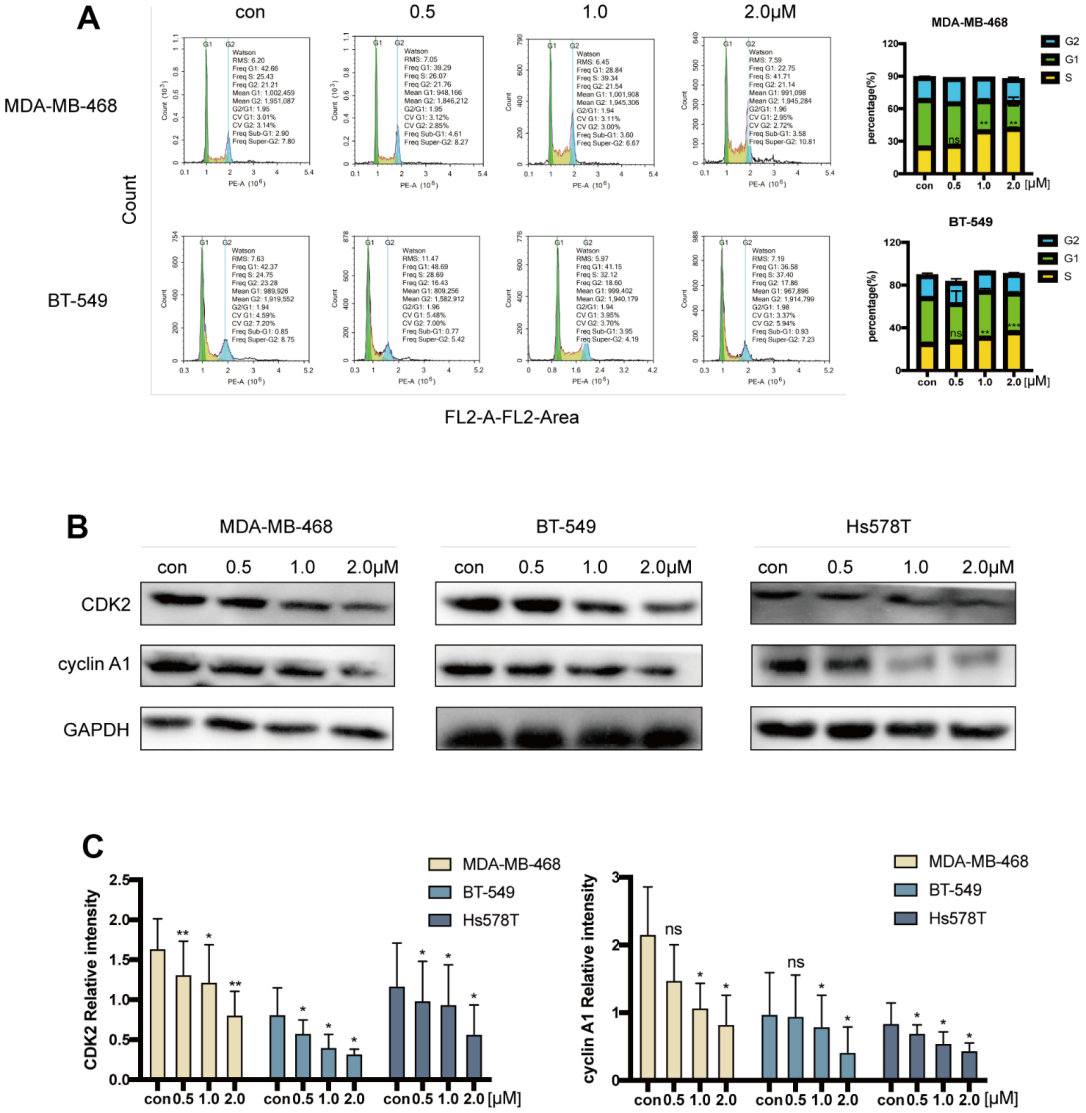
**Acetyl-cinobufagin induced S/G2 cell cycle arrest.** (**A**) Cell cycle distribution in MDA-MB-468 and BT-549 cells treated with acetyl-cinobufagin treatment evaluated by flow cytometry. (**B**, **C**) The immunoblotting analysis was performed to determine the levels of cyclin A1 and CDK2 after treatment with acetyl-cinobufagin. *p < 0.05; **p < 0.01; ***p < 0.001 compared with the control based on Student’s t-test.

By determining the percentage of cells in the S to G2 phase transition by using flow cytometric analysis on MDA-MB-468 and BT-549 cells injected with acetyl-cinobufagin for 12 hours, the acetyl-cinobufagin-induced retardation of cell cycle progression was assessed. Acetyl-cinobufagin significantly promoted the delay of cell cycle progression in the S/G2 transition from one phase to another when compared to the normal control (vehicle) group (Figure 4A). We examined CDK2 and cyclin A1’s expression using an immunoblotting technique because these are two well-known essential proteins for S-phase arrest. The outcomes demonstrated that acetyl-cinobufagin administration significantly reduced the expression of CDK2 and cyclin A1 in human TNBC cells (Figure 4B, 4C). These results suggest that acetyl-cinobufagin’s suppressive impact on the proliferation of human TNBC cells may be partially explained by the activation of cell cycle progression retardation during the S-to-G2 transition of phases.

### Acetyl-cinobufagin inhibits EMT via STAT3 signaling

**Figure 5:**
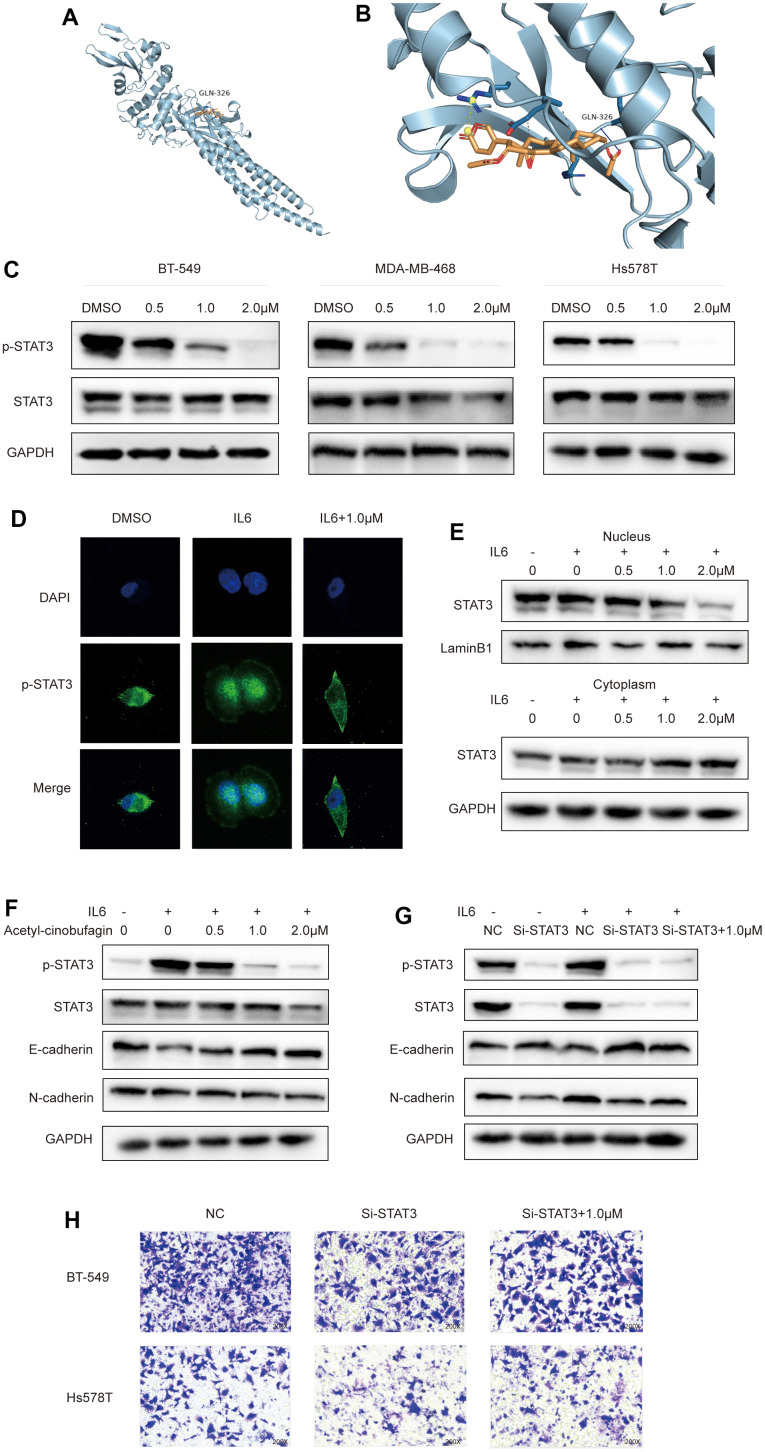
**Acetyl-cinobufagin inhibits EMT through the STAT3 signaling pathway.** (**A**, **B**) Molecular docking of acetyl-cinobufagin to the STAT3 binding sites. (**C**) Expression of proteins involved in the STAT3 signaling pathway as detected by Western blot analysis. (**D**) The subcellar location of STAT3 was examined by immunofluorescence staining and confocal laser scanning microscopy (CLSM) in BT-549 cells. (**E**) The levels of STAT3 protein in the cytoplasm and nuclei were determined by immunoblotting analysis after extraction from BT-549 cells. (**F**) BT-549 cells treated with IL6 for 30 min after treatment with acetyl-cinobufagin for 10-20 h; expression levels of proteins associated with STAT3 and EMT signaling pathways as detected by Western blot analysis. (**G**) STAT3 knockdown in BT-549 cells treated with control, IL6 or IL6 + acetyl-cinobufagin (1.0 μM), expression levels of proteins related to STAT3 and EMT signaling pathways as detected by Western blot analysis. (**H**) Evaluation of metastatic abilities of BT-549 and Hs578T after knockdown of STAT3.

The molecular docking results revealed that acetyl-cinobufagin can occupy the STAT3 binding sites and form hydrogen bonding with the GLN-326 residue (Figure 5A, 5B). Given that a derivative of cinobufagin has been previously found to exhibit anticancer activity by suppressing the STAT3 signaling pathway, we hypothesized that acetyl-cinobufagin might impact STAT3 phosphorylation (P-STAT3). We performed Western blot analysis to investigate whether acetyl-cinobufagin impacts the expression of P-STAT3 in MDA-MB-468, BT-549, and Hs578 cells and found that acetyl-cinobufagin time- and dose-dependently regulates the expression of P-STAT3 (Figure 5C, and Supplementary Figure 1E). Additionally, P-STAT3 was shown to be restricted in the cytoplasm and later translocated to the nucleus in response to IL6 activation, as shown by accumulating more of the green fluorescence protein in the cell nuclei, according to immunofluorescence staining. We also found that such IL6-induced translocation to the nucleus can be reversed by treatment with acetyl-cinobufagin (Figure 5D). These findings suggested that acetyl-cinobufagin reduced IL6-induced STAT3 nuclear translocation. We also examined the amounts of nuclear and cytoplasmic STAT3 proteins following IL6 therapy. After 30 minutes of IL6 stimulation, compared to unstimulated cells, nuclear STAT3 protein levels increased but cytoplasmic STAT3 protein levels decreased. We also found that acetyl-cinobufagin suppressed IL6-induced STAT3 nuclear translocation in a dose-dependent manner (Figure 5E). These researches indicate that acetyl-cinobufagin suppresses STAT3 signaling and interferes with the STAT3 nuclear translocation.

Past research has revealed that EMT occurs downstream of the STAT3 signaling pathway. As a result, we looked into whether acetyl-cinobufagin could reduce carcinogenesis and migration by blocking the STAT3 signaling pathway. We discovered that when TNBC cells were treated with IL6 (20 ng/mL) for 30 min, P-STAT3 levels significantly increased in comparison to the control group. Additionally, N-cadherin’s expression increased while E-cadherin’s expression dropped. Such effects were suppressed by treatment with acetyl-cinobufagin, which reduced P-STAT3 and inhibited EMT (Figure 5F). Through Western blot analysis, which revealed that STAT3 si-RNA3 had the largest repressive effect on STAT3, we also created and chose si-STAT3, a siRNA with the greatest inhibitory effect against STAT3 (Supplementary Figure 1F). However, as can be shown in Figure 5G, the results of the study demonstrated that the STAT3 siRNA3 decrease of STAT3 expression had no appreciable impact on EMT. These results were further verified by the knockdown of STAT3 in TNBC cells before the treatment with 1.0 μM acetyl-cinobufagin. As shown in Figure 5H, the knockdown of STAT3 had no noticeable effect on the invasive and metastatic properties of the TNBC cells. Additionally, Western blot analysis was done to validate the acetyl-cinobufagin impact via STAT3 overexpression. The results revealed that acetyl-cinobufagin regulates EMT by suppressing phosphorylation of overexpression of STAT3 (Supplementary Figure 1H). In general, acetyl-cinobufagin exerted a potent antitumor effect by suppressing EMT in TNBC cells through the STAT3 signaling pathway.

### Acetyl-cinobufagin inhibits the growth of the TNBC xenograft model

**Figure 6:**
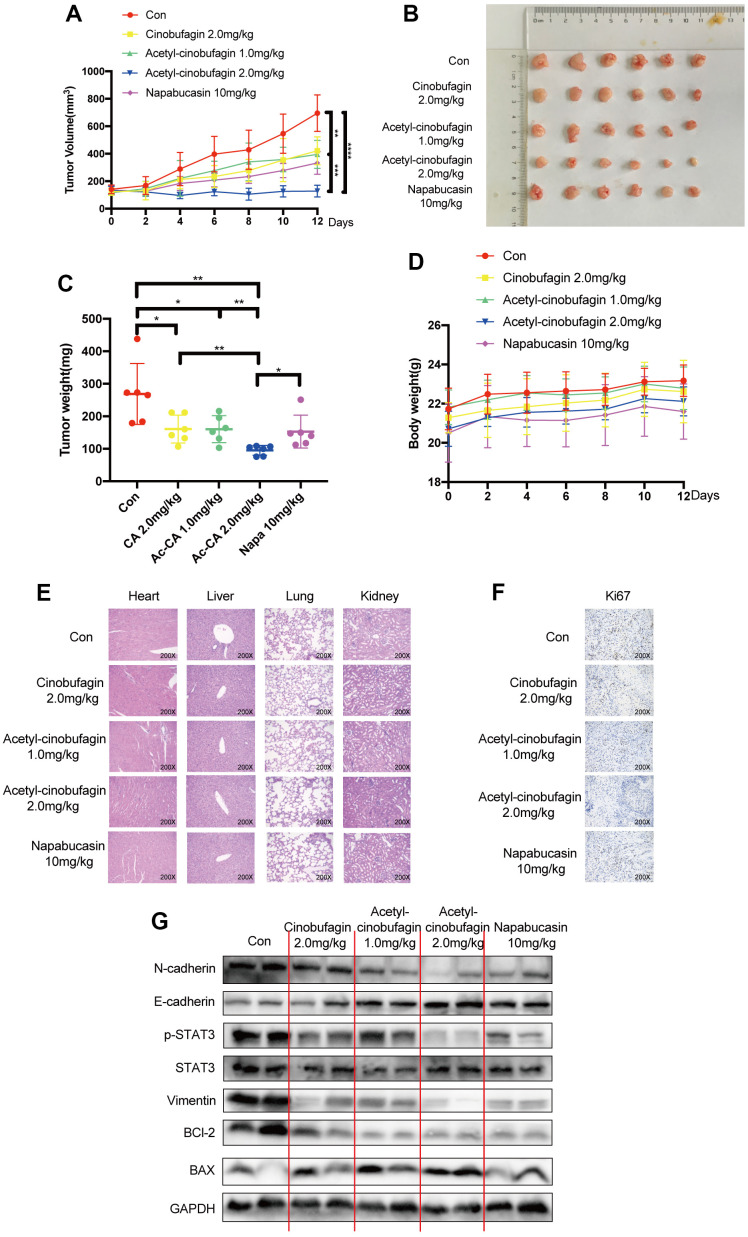
**The role of acetyl-cinobufagin in growth suppression of TNBC *in vivo* in a human xenograft model established by orthotopically injecting BT-549 cells.** (**A**) BALB/c animals were divided into Control, napabucasin 10 mg/kg, cinobufagin 2.0 mg/kg, acetyl-cinobufagin 1.0 mg/kg, and acetyl-cinobufagin 2.0 mg/kg groups. The cancer volumes were recorded every 2 days and the curve was eventually drawn. (**B**) Gross view of xenograft tumor tissue. (**C**) Ultimately, the animals were sacrificed, and the cancer tissue was harvested and weighed. (**D**) The weight of the animals was recorded during the assay. (**E**) No histology abnormity was detected by H&E staining of the main organs in the 5 animal groups. (**F**) Immunohistochemical staining images of the expression of the cellular proliferative biomarker Ki67 in cancer samples. (**G**) Expression of STAT3- and EMT-related proteins detected by Western blot analysis. Two-way analysis of variance (ANOVA) was used to study the tumor volume and the Student’s t-test was used for others with p-value significant codes: *p < 0.05; **p < 0.01; ***p < 0.001; ****p < 0.0001.

We also looked into the *in vivo* suppression of STAT3 and EMT by acetyl-cinobufagin. To test the inhibitory effect of acetyl-cinobufagin on TNBC cells *in vivo*, a human xenograft model was established by orthotopically injecting BT-549 cells into the mammary fat pad. On alternate days, we administered intraperitoneal injections of napabucasin (10 mg/kg; positive control), cinobufagin (2.0 mg/kg), or various concentrations of acetyl-cinobufagin (1.0 or 2.0 mg/kg) to the mice. The nude mice’s human xenograft tumor growth curves demonstrated a significant reduction in tumor growth and tumor volume in the treated animals (Figure 6A, 6B). The tumor weight was also decreased concurrently (Figure 6C). Throughout the therapy period, there was no discernible difference in body weight (Figure 6D). The primary organs exhibiting morphogenetic processes were H&E stained, and the results showed no change (Figure 6E). These findings disproved any cytotoxicity in mice treated with acetyl-cinobufagin, demonstrating the drug’s efficacy *in vivo*. By analyzing the expression of Ki67 immunohistochemically, we looked at the impact of acetyl-cinobufagin on the growth of cancer. As a result, the gene expression level of Ki67 decreased (Figure 6F). These results demonstrated that acetyl-cinobufagin significantly inhibited TNBC cell proliferation *in vivo* via the STAT3 signaling pathway. Acetyl-cinobufagin inhibited STAT3 signal transmission and EMT, according to the immunoblotting assay (Figure 6G). Additionally, these findings demonstrated that acetyl-cinobufagin is a possible therapeutic option for TNBC. Our study provides inspiration for the development of tumor therapy by suppressing the migratory behavior of TNBC cells through the regulation of STAT3 signaling.

## DISCUSSION

Cinobufagin, a natural compound derived from toad venom, is one of the primary bioactive constituents found in cinobufotalin injection, a widely utilized treatment for intermediate and advanced cancers [25]. However, the rapid metabolism of cinobufagin, toxicity, insolubility and short half-life have limited its clinical use [23]. In this study, we used a similar approach as that used for aspirin and acetyl-bufalin to acetylate cinobufagin, which improved its curative effect without its toxic side effects. For example, acetyl-cinobufagin exhibited better tolerance in animals treated with a high-dose compared to treated with cinobufagin (Figure 1D, 1E). Moreover, acetyl-cinobufagin had a better therapeutic effect than cinobufagin (Figure 6A–6C). In addition, we used TNBC cells to determine the functional role of acetyl-cinobufagin. It was found the lowest IC_50_ of acetyl-cinobufagin reached 1.12 μM against MDA-MB-468 cells. Additionally, it has been demonstrated that acetyl-cinobufagin promotes cell death while reducing TNBC cell proliferation. Acetyl-cinobufagin also delayed the S/G2 phase transition in TNBC cells, just like cinobufagin did [26]. Additionally, we confirmed acetyl-cinobufagin’s specific anticancer processes, long-term steady performance, and little toxic effects. Additionally, we also verified the distinct anticancer mechanisms, as well as the long-term stable efficacy and low toxicity of acetyl-cinobufagin. Together, our results suggest that acetyl-cinobufagin had a more effective anticancer activity than cinobufagin.

EMT has been strongly associated with cancer development and metastasis in a variety of tumor types [27]. Numerous signaling pathways and transcription factors are involved, including the JAK/STAT, TGF-, PI3K/AKT, Ras/MAPK, Wnt, Notch, and Hedgehog signaling pathways [28, 29]. EMT involves several events, including the loss of epithelial polarity, the separation of the basement membrane, and the acquisition of mesenchymal characteristics, but not those of migration and invasion characteristics [30]. One of the most prominent hallmarks of TNBC is EMT, a conservative development process often hijacked by cancer cells to enhance their migration and invasion abilities [31]. It is widely established that IL6 promotes TNBC invasion and migration [32]. In contrast to vimentin and protein N-cadherin, the expression of the protein E-cadherin was shown to be higher in this study. Therefore, our findings indicate that acetyl-cinobufagin decreased the migration of TNBC cells by inhibiting EMT.

In the tumor microenvironment (TME), the IL6/JAK/STAT3 signaling pathway promotes the proliferative, survival, invasive, and metastatic activities of cancer cells, and strongly inhibits the anticancer immune response [33]. Signaling through the IL6/JAK/STAT3 pathway after activation by the binding of the IL-6 family of cell factors (IL6 and IL-11) to the receptors has been shown in BC progression [34]. After IL6 interacts with its receptor, Tyr-705 in STAT3 is phosphorylated to promote STAT3, which causes nuclear translocation, recognition of STAT3-specific DNA binding sites, and stimulation of targeted genetic transcription [35]. The IL6R/STAT3/miR-34a feedback loop was found in the breast, and it was found that EMT, invasiveness, and metastatic activity required an activated IL6R/STAT3/miR-34a feedback loop [36]. In this study, by suppressing STAT3 phosphorylation and nuclear translocation, acetyl-cinobufagin modulated the STAT3-mediated transcriptional activation. Cells with knocked-down STAT3 were still capable of migrating and invading, even after acetyl-cinobufagin treatment. In conclusion, our results suggest that acetyl-cinobufagin, which suppresses EMT in BC by focusing on the STAT3 signaling pathway, is a possible candidate for tumor therapy.

According to this study’s comprehensive analysis, acetyl-cinobufagin suppresses TNBC both *in vitro* and *in vivo* by preventing STAT3 from being stimulated. The results of this investigation and the contributions of earlier research point to the therapeutic efficacy of acetyl-cinobufagin. However, more research is needed to achieve the optimization of acetyl-cinobufagin for its clinical application.

## MATERIALS AND METHODS

### Cell culture

Various human BC cell lines (Hs578T, BT-549, MDA-MB-468, and MDA-MB-231) and the commonly used normal (non-tumorigenic) human mammary epithelial cells (MCF-10A) from the Cell Bank of the Shanghai Chinese Academy of Sciences (Shanghai, China) were used for the research in this study. The Dulbecco’s modified Eagle’s medium (DMEM; Invitrogen, Carlsbad, CA, USA) was used to cultivate the Hs578T and MDA-MB-231 cell lines. It was supplemented with 10% Gibco fetal bovine serum (FBS; Thermo Fisher Scientific Inc., Waltham, MA, USA) and 1% penicillin/streptomycin (Solarbio, Beijing, China). The Gibco Roswell Park Memorial Institute (RPMI)-1640 medium (Thermo Fisher Scientific Inc.) was used to cultivate the BT-549 cell line and supplemented with 10% Gibco FBS. Gibco Leibovitz’s L-15 medium (Thermo Fisher Scientific Inc.) was used to cultivate the MDA-MB-468 cell line, and 10% Gibco FBS was added as a dietary supplement. Gibco DMEM/F12 media (Thermo Fisher Scientific Inc.) supplemented with 10% Gibco FBS was used to cultivate the MCF-10A cell line. Except the MDA-MB-468 cell line, all cell lines were kept in an incubator at 37° C, with a humidified environment and 5% CO2; the latter was not present.

### Methyl thiazolyl tetrazolium (MTT) cytotoxicity analysis

The cytotoxicity (loss of viable cells) of the test substances, cinobufagin, and acetyl-cinobufagin, was assessed in human BC cell lines and normal breast cells using the MTT assay. MTT analysis was performed as follows. 8 × 10^3^ cells were inserted into 96-well plates, and the plates were then left in the incubator overnight. Following treatment with the proper test chemical, after treatment with the appropriate test compound, 25 μL of the MTT reagent was then put into each well, and the plates underwent an additional 4 hours of incubation at 37° C. Afterward, the produced formazan crystals were dissolved in 50 μL of dimethyl sulfoxide (DMSO) after the growth media was removed, and the optical density (OD) of each well was then determined at 490 nm using a Microplate Reader. Cell activity = (mean OD value of treated wells/mean OD value of vehicle control wells) 100% was used to calculate the cytotoxicity. GraphPad Prism 8.0 software (GraphPad Software Inc., San Diego, CA, USA) was used to calculate the half maximum inhibitory concentration (IC50) values.

### Acute toxicity test

Thirty female NIH 8-week-old mice were distributed at random to one of three groups—vehicle, cinobufagin (30 mg/kg), or acetyl-cinobufagin (30 mg/kg)—of ten mice each for the acute toxicity assay. On the first day, the relevant chemical was intraperitoneally delivered to the mice in each group. All of the animals were housed in a 12-hour light/dark cycle at a temperature of 25° C with unlimited access to food and water. The death rate and animal weight were measured as outcomes for each group daily for 14 days. After the experiment, cervical dislocation was used to kill every animal.

### Colony assay

Six-well plates were used, and 1,500 cells were planted into each well. The cells were grown in an incubator at 37° C with a humidified atmosphere and 5% CO2 for 6–8 days following treatment with medications or DMSO for 12 hours. The cells were then stained for 15 minutes at 24° C with 0.1% crystal violet and preserved with 5% paraformaldehyde (PFA). A camera was used to record every picture of labeled cells. Three duplicates of each experiment were carried out.

### Wound-healing migration assay

The wound healing experiment was used to evaluate the cell migratory capacity. Once the cells had grown to between 80 and 90 percent confluence in 6-well plates, a long scratch wound was created on the cell monolayer using a sterile 10-μL pipet tip. Cells were continued to culture in serum-free media with vehicle control or acetyl-cinobufagin (0.25, 0.5, 1.0 μM) for 48 hours after the wells had been rinsed with phosphate-buffered saline (PBS) to remove the unbound cells. Using an inverted microscope attached to an imaging system (Leica Biosystems GmbH, Wetzlar, Germany), images were taken at 0, 24, and 48 h to track the migration of cells to the wound area.

### Transwell invasion assay

The invasion test was conducted using Corning® Transwell® polycarbonate membrane cell culture inserts (#3422; Corning Inc., Corning, NY, USA). Cells (MDA-MB-468, BT-549, and Hs578T) were collected, washed with PBS, and then mixed in the appropriate serum-free media before being seeded at a density of 3×10^4^ cells per well onto the upper side of the Transwell inserts in the upper chamber. 10% FBS was added to a 600-μL volume of the equivalent medium in the lower chamber. After 24 hours, the cells on the Transwell inserts’ upper surface were taken out by using cotton swabs to clean the insert’s upper side. After that, PFA was used to attach the cells to the lower surface of the inserts, and 0.4% crystal violet was used to stain them. The next step was to count positively labeled cells in three random fields for each cell line examined using a microscope at a magnification of 20.

### Immunofluorescence staining

On fluorescent slides of glass, BT-549 cells were plated and cultivated while being treated with the appropriate substance. After that, cells were permeabilized in PBS containing 0.5% Triton X-100, washed four times with PBS, and blocked with 1% bovine serum albumin (BSA) in PBS for 60 minutes. Cells were then preserved with 4% PFA. The relevant primary antibody (P-STAT3, E-Cadherin, N-Cadherin, or Ki67) was then left to be incubated overnight at 4° C with cell slides (Thermo Fisher Scientific Inc., Waltham, MA, USA), then incubated the cell slides with an anti-rabbit secondary antibody or an anti-mouse secondary antibody conjugated with Alexa Fluor® 488, and the nuclei were counterstained with 4’,6-diamidino-2-phenyl-indole dihydrochloride (DAPI) (Beyotime Biotechnology, Shanghai, China) in the dark. The labeled cells were then observed by confocal laser scanning microscopy using the Zeiss LSM 710 Confocal Laser Scanning Microscope (Carl Zeiss AG, Oberkochen, Germany) to record the images. The cell slides were then rinsed once with PBS and sealed with an anti-fluorescence quenching sealing solution.

### Hoechst 33342 staining

MDA-MB-468, BT-549, and Hs578T cells were stained with Hoechst 33342 using a kit from the Beyotime Institute of Biotechnology in Shanghai, China, to examine the distinctive morphological changes that occur during apoptosis. 3×10^5^ mL of cells were planted into 6-well plates, and either DMSO (Control) or acetyl-cinobufagin (0.5, 1.0, 2.0 μM) was applied to the cells. Cells were incubated for 24 hours before being rinsed with PBS, fixed with 4% PFA for 15 minutes, and then again dealt with PBS. Apoptotic nuclei were then stained by incubating cells for 20 minutes in the dark at 37° C with Hoechst 33342 (v_Hoechst 33342_/v_PBS_ at 1:1000) ways. After capturing images using a Leica fluorescence microscope (Leica Biosystems GmbH), cells were finally rinsed with PBS, with an antifade mounting approach, and apoptotic cells were detected.

### Apoptosis assay

The BD PharmingenTM Annexin V-fluorescein isothiocyanate (FITC)/PI apoptosis detection kit (#556,547; BD Biosciences, Franklin Lakes, NJ, USA) was used for the flow cytometry-based cell apoptosis assay. 6-well plates of cells were seeded, and they were cultivated with or without acetyl-cinobufagin at the appropriate doses. Following treatment, cells were collected, resuspended, and tested for apoptosis using the Annexin V-FITC/PI assay per the manufacturer’s instructions. In a nutshell, cells were resuspended in 500 μL of binding buffer and stained by incubating with 5 μL of PI for 5 min and 5 μL of FITC-conjugated Annexin V for 15 min while on ice and in the dark. On a BD Accuri C6 Flow Cytometer (BD Biosciences), information was gathered using flow cytometry from a total of 20,000 fluorescently labeled cells/sample. The flow cytometry data were analyzed using the FlowJo software (FlowJo, Ashland, OR, USA) to calculate the proportion of early (Q3, Annexin V+/PI+) and late (Q2, Annexin V+/PI+) apoptotic cells, as well as the overall apoptotic cells.

### Cell cycle assay

MDA-MB-468 and BT-549 cells were seeded onto 6-well plates and allowed to grow for 12 hours. Following that, the cells were exposed to either DMSO (control) or different concentrations (0.5, 1.0, and 2.0 M) of acetyl-cinobufagin for 24 hours. After the incubation period, the cells were harvested and treated with a solution of 70% ethanol chilled at -20° C. Subsequently, the cells underwent enzymatic digestion using RNase at a concentration of 0.1 mg/mL for 10 minutes at 37° C. To analyze the cell cycle, the cells were stained with propidium iodide (PI) at a concentration of 10 g/mL. The DNA content of each sample was then measured using a FACSCalibur flow cytometer (BD Biosciences).

### Western blot analysis

On 6-well plates, MDA-MB-468 and BT-549 cells were plated, cultured for 12 hours, and then given DMSO or acetyl-cinobufagin (0.5, 1.0, 2.0 M) for 24 hours. After incubation, cells were collected, addressed with ice-cold 70% ethanol at -20° C, digestion with 0.1 mg/mL RNase for 10 min at 37° C, and labeled with 10 g/mL PI. Data on the DNA content of every specimen were then recorded for cell cycle analysis using a FACSCalibur flow cytometer (BD Biosciences). Subsequently, the proteins were separated on a precast 10% sodium dodecyl sulfate-polyacrylamide gel electrophoresis (SDS-PAGE) gel (BioRad Laboratories, Hercules, CA, USA). The proteins were then separated using SDS-PAGE (BioRad Laboratories, Hercules, CA, USA), a precast 10% sodium dodecyl sulfate-polyacrylamide gel electrophoresis gel. The separated proteins were then put on 0.45-m polyvinylidene fluoride (PVDF) membranes from EMD MilliporeSigma in Burlington, MA, USA. The membranes were blocked for 1 hour at room temperature in 5% skim milk in Tris buffer saline with 0.1% tween-20 (TBST), and then the blots were treated with the appropriate primary antibodies overnight at 4° C. The blots were subsequently rinsed with PBS and exposed to the appropriate horseradish peroxidase (HRP)-conjugated secondary antibodies (1:5,000, Abcam, Cambridge, UK) for 1 hour at room temperature. The band patterns were then observed with the electrochemiluminescence (ECL) detection equipment (Thermo Fisher Scientific Inc.) by the instructions provided by the maker after washing the blots. The National Institutes of Health (NIH), Bethesda, MD, USA, produced the GAPDH protein and employed it as a loading control while measuring the relative intensity for each labeled band. No fewer than three times for each of the tests were run. Primary antibodies used in this study were as follows: E-cadherin (1:5,000, 20874-1-AP, Proteintech, Wuhan, China); N-cadherin (1:2,000, 22018-1-AP, Proteintech); Vimentin (1:2,000, 10336-1-AP, Proteintech); P-STAT3 (1:2,000, ab76315, Abcam); STAT3 (1:1,000, 12640S, Cell Signaling Technology (CST) Inc., Danvers, MA, USA).

### Extraction of cytoplasmic and nuclear proteins

In a 10-cm plate of 80–90% confluent BT-549 cells, acetyl-cinobufagin (0.5, 1.0, and 2.0 M) was applied. The cells were put on ice as soon as they had been stimulated with IL6 for 0.5 hours. The NE-PERTM Nuclear and Cytoplasmic Extraction Kit (Cat# 78833; Thermo Fisher Scientific Inc.) was used to extract the proteins from the nucleus and cytoplasm. The immunoblotting study was carried out to find out whether related proteins were expressed.

### Transfection

Negative-control siRNAs (si-NC) and STAT3 small interfering RNAs (STAT3 si-RNAs) were created using synthetic small interfering RNAs (si-RNAs). The BT-549 cells were momentarily implanted with the siRNAs. The following were the STAT3 si-RNAs: Three STAT3 siRNAs have been identified: STAT3 si-RNA1 STAT3-Homo-978, 5-GCAACAGAUUGCCUGCAUUTT-3, STAT3 si-RNA2 STAT3-Homo-398, and STAT3 si-RNA3 STAT3-Homo-1070, 5-CCCGUCAACAAAUUAAGAATT-3. To overexpress STAT3, the full-length STAT3 sequence was cloned into the p-CMV (Genechem, Shanghai, China) expression vector. BT-549 has been transgenic with STAT3-pCMV using Invitrogen’s Lipofectamine 3000. The cells were gathered for further processing after 48 hours.

### Molecular docking

Acetyl-cinobufagin was subjected to molecular docking analysis with the binding sites of STAT3 using AutoDock Vina 4.2.6 software (https://ccsb.scripps.edu/autodock/). The crystal structure of STAT3 obtained from the Protein Data Bank served as the basis for the docking study. For the preparation of input data, AutoDock Tools 1.5.6, a Graphical User Interface program, was utilized to create the ligand and receptor configurations. During the docking process, the receptor (STAT3) was considered rigid, while the ligand (acetyl-cinobufagin) was treated as flexible.

### Animal model

The female BALB/c athymic nude mice used in this study were kept in the experimental animal department of WMU and their use was approved by the ethical committee of WMU. The nude mice were subcutaneously injected with BT-549 cells (5 × 10^6^) which were diluted by mixing with the appropriate quantity of Matrigel (BD Biosciences) and PBS (orthotopically). After 7-10 days, when the tumor measured ~100 mm^3^, the animals were assigned into different groups for the assays (control group, positive control group receiving napabucasin at 10 mg/kg, treatment group A receiving acetyl-cinobufagin at 1.0 mg/kg, and treatment group B receiving acetyl-cinobufagin at 2.0 mg/kg, treatment group C receiving cinobufagin 2.0 mg/kg). There were more than 6 mice in every group, and treatment was administered by intraperitoneal injection. The tumor size and body weight variations were recorded every other day for 12 days. After completion of the experiments, mice were sacrificed, and cancer tissue and organs, such as heart, liver, kidneys, and lungs were harvested and used for subsequent immunoblotting assays and hematoxylin and eosin (H&E) staining.

### Statistical analysis

To confirm the study’s repeatability and dependability, every study was separately carried out no fewer than three times. GraphPad Prism 8.0 software was used to perform statistical analysis of the data. The Student's t-test was used to examine the variation between experimental groups. If the p-value was less than 0.05 for all analyses, the difference was deemed significant and showed proof of the successful treatment with acetyl-cinobufagin.

### Consent for publication

To publicize this study and relevant figures, the researchers distributed written informed consent to patients.

### Availability of data and materials

Raw information supporting the concluding remarks here are available on the major electronic information storage system pertaining to The First Affiliated Hospital of Wenzhou Medical University, and access can be offered in line with the authors.

## Supplementary Material

Supplementary Figure 1
