# Supplementary material for: Acetyl-cinobufagin suppresses triple-negative breast cancer progression by inhibiting the STAT3 pathway
Source: Aging (Albany NY). 2023 Aug 28;15(16):8258–74. doi: 10.18632/aging.204967 (PMC10497018; doi:10.18632/aging.204967)
Supplement: Supplementary Figure 1 [file aging-15-204967-s001.pdf]

SUPPLEMENTARY FIGURE

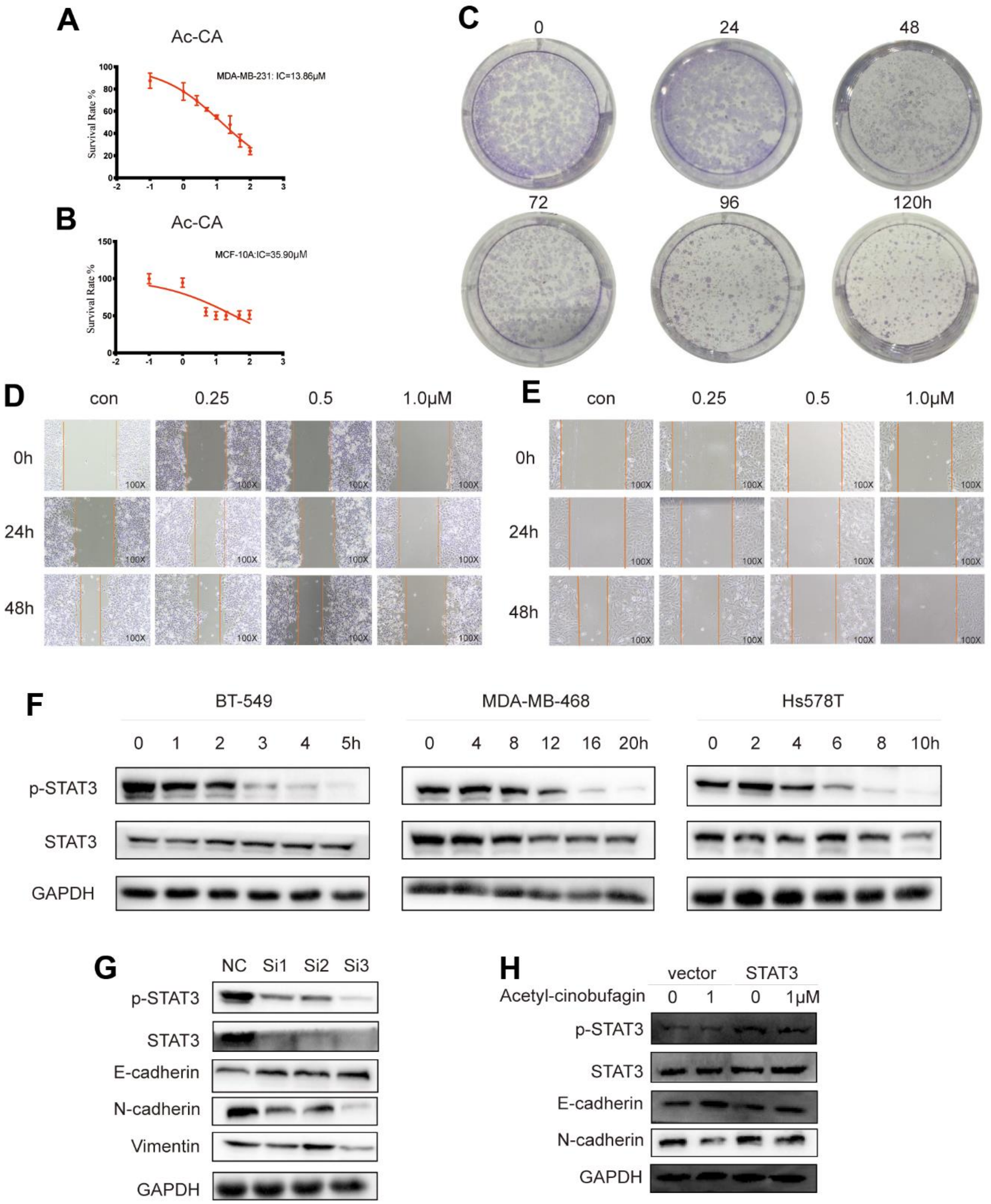

Supplementary Figure 1. (**A**, **B**) The suppression rate was determined using the MTT assay. (**C**) Colony forming analysis was completed using the Hs578T with the different duration of action of acetyl-cinobufagin. (**D**, **E**) Wound healing assay was performed using the MDA-MB-468 and Hs578T cell lines with/without acetyl-cinobufagin treatment. (**F**) Expression of proteins associated with the STAT3 signaling pathway as detected by Western blot analysis. (**G**) Selection of the si-STAT3 with maximum inhibitory activity by Western blot analysis in BT-549 cells. (**H**) Overexpression of STAT3 in BT-549, expression levels of proteins associated with STAT3 and EMT signaling pathways as detected by Western blot analysis.
